# Efficient and Ambient-Air Processed Perovskite Solar Cells Enabled by Adamantanethiol as a Dual-Action Modulator

**DOI:** 10.3390/molecules31193485

**Published:** 2026-09-30

**Authors:** Fujie Cai, Xin Deng, Yuwei Xia, Pengwei Wan, Yong Du, Xiangjian Cheng, Yifeng Li, Jiahao Deng, Yintong Pu, Jingwen Huang, Yong Li, Chen Sun, Weifan Luo

**Affiliations:** 1 Sichuan Rangtang Huadian New Energy Co., Ltd., Chengdu 610041, China; 2 Optoelectronic Sensor Devices and Systems Key Laboratory of Sichuan Provincial Universities, College of Optoelectronic Engineering (Chengdu IC Valley Industrial College), Chengdu University of Information Technology, Chengdu 610225, China; 3 Sichuan Meteorological Optoelectronic Sensor Technology and Application Engineering Research Center, Chengdu University of Information Technology, Chengdu 610225, China; 4 Information Materials and Device Applications Key Laboratory of Sichuan Provincial Universities, Chengdu University of Information Technology, Chengdu 610225, China; 5 Dongguan Key Laboratory of Micro-Nano Optical Field Manipulation and Sensing, School of Physical Sciences, Great Bay University, Dongguan 523000, China; 6 State Key Laboratory of Structural Analysis, Optimization and CAE Software for Industrial Equipment, National Engineering Research Center for Advanced Polymer Processing Technology, Zhengzhou University, Zhengzhou 450001, China

**Keywords:** perovskite solar cells, 1-adamantanethiol, charge carrier dynamics, terahertz time-domain spectroscopy

## Abstract

This work presents a dual-modulation strategy using 1-adamantanethiol (ADT), a rigid cage-like molecule, to simultaneously enhance the efficiency and stability of inverted perovskite solar cells. In contrast to conventional flexible small molecule modulators, ADT features a highly rigid three-dimensional adamantane skeleton that enables the formation of a stable steric barrier layer on the perovskite surface. Meanwhile, its terminal –SH group exhibits strong coordination affinity with undercoordinated Pb^2+^ ions, providing effective chemical passivation of surface defects. Beyond defect healing, the ADT treatment further modulates the interfacial energy-level alignment and optimizes charge carrier extraction and transport, as systematically verified by a suite of advanced optical characterizations, including transient absorption (TA) spectroscopy and terahertz time-domain spectroscopy (THz-TDS). Taken together, these techniques confirm accelerated carrier dynamics and suppressed recombination losses upon ADT passivation. All solution-processing steps were conducted in ambient air (20–25 °C, 40–60% RH), where the hydrophobic ADT layer protects the perovskite from moisture. As a result, the inverted perovskite solar cells fabricated entirely in ambient air incorporating ADT achieve a power conversion efficiency of 23.9%, alongside significantly enhanced operational stability, as evidenced by maintaining superior performance during 400 h of maximum power point (MPP) tracking under continuous illumination.

## 1. Introduction

Over the past decade, organic–inorganic hybrid perovskite solar cells (PSCs) have attracted tremendous interest in the photovoltaic community, driven by their exceptional power conversion efficiencies and low fabrication costs [1]. Structurally, these materials conform to the ABX_3_ perovskite lattice, with the A position occupied by methylammonium (MA^+^), formamidinium (FA^+^), or cesium (Cs^+^), and the B position by divalent lead (Pb^2+^), coordinated within a halide anion octahedron (X = I^−^, Br^−^, Cl^−^; Figure 1a) [1,2]. Despite their superior optoelectronic characteristics, the widespread deployment of PSCs is still constrained by persistent issues, namely poor resistance to environmental factors like moisture and oxygen, severe ionic migration during device operation, and a noticeable drop in efficiency upon upscaling to larger areas [3,4,5]. To overcome these hurdles, numerous strategies have been put forward, ranging from additive incorporation in precursor solutions [6] and dimensionality engineering with 3D/2D heterostructures, to the adoption of self-assembled monolayers (SAMs) [2,7]. Among these routes, interfacial passivation has emerged as a particularly attractive approach, owing to its straightforward implementation and remarkable effectiveness in reducing non-radiative recombination losses [8]. Among the interfacial modulators, thiol-functionalized molecules have emerged as effective multifunctional modifiers for perovskite solar cells [9]. The sulfhydryl (–SH) group, acting as a soft Lewis base, strongly coordinates with undercoordinated Pb^2+^ to passivate deep-level defects and suppress non-radiative recombination [10,11]. Beyond defect passivation, thiols can modulate crystallization kinetics, relieve residual strain, and generate interfacial dipoles that optimize energy-level alignment [10,11,12]. Their redox activity further enables scavenging of corrosive iodine species, mitigating interfacial degradation. Depending on their location, at charge-transport layer interfaces, within the perovskite bulk, or at the electrode, thiols provide tailored functions including chemical passivation, physical encapsulation, and enhanced mechanical adhesion, together boosting both efficiency and stability [10,11,12,13]. These unique attributes make thiol-based strategies a promising route toward stable and efficient PSCs. Among the diverse thiol-based modifiers developed for perovskite interfaces, most investigations have centered on flexible alkyl or aryl thiols. In parallel, rigid cage-like molecular skeletons, particularly those featuring an adamantane core, have recently garnered increasing attention in perovskite optoelectronics [14]. For instance, it has been demonstrated that adamantane derivatives can effectively mitigate interfacial defect states, thereby enhancing both the photovoltaic performance and operational stability of perovskite solar cells [14]. Furthermore, their successful integration into formamidinium-based layered two-dimensional (2D) perovskites has also been reported, highlighting the compatibility of this bulky hydrophobic scaffold with complex perovskite lattices [15]. Despite these promising advances, the potential of rigid thiol-functionalized cage molecules remains largely untapped in this field. Unlike previously reported adamantane-based systems, which primarily rely on their bulky hydrophobic nature for protection, molecules leverage the unique combination of a rigid adamantane backbone with multiple terminal thiol groups, the sulfhydryl-terminated 1-adamantanethiol (ADT) has received limited attention. The rigid three-dimensional structure of ADT is expected to offer distinct steric shielding and physical encapsulation beyond the chemical modulation provided by conventional flexible thiols, making it a promising candidate for synergistic defect healing and stability enhancement.

Herein, we implement 1-adamantanethiol (ADT) as a rigid cage-like interfacial modifier for inverted perovskite solar cells. Ultraviolet photoelectron spectroscopy (UPS) and Kelvin probe force microscopy (KPFM) measurements reveal that ADT treatment effectively modulates the surface energy levels and reduces the contact potential difference, facilitating more efficient hole extraction at the perovskite/hole-transport-layer interface. Furthermore, transient absorption spectroscopy (TAS) and terahertz time-domain spectroscopy (THz-TDS) confirm accelerated carrier transport and suppressed recombination losses upon ADT modulation. As a result, the ADT-modified inverted devices fabricated in ambient air achieved a champion power conversion efficiency of 23.9%, alongside substantially enhanced operational stability under damp-heat conditions compared to the control devices. These findings highlight rigid thiol molecules such as ADT as promising candidates for advancing the efficiency and durability of perovskite photovoltaics.

## 2. Results

### 2.1. Molecular Design

The molecular design of 1-adamantanethiol (ADT) is rationalized by its distinctive combination of electronic structure and steric configuration. As shown in Figure 1b, the electrostatic potential (ESP) map reveals that the negative electrostatic potential is predominantly localized around the terminal thiol (–SH) group, indicating its strong electron-donating nature and high affinity for electrophilic species such as undercoordinated Pb^2+^ ions [12,16]. In contrast, the adamantane cage skeleton exhibits a relatively neutral and uniform electrostatic distribution, which minimizes unwanted charge interactions with the perovskite lattice [14,15]. The calculated frontier molecular orbitals (Figure 1c) show that the highest occupied molecular orbital (HOMO) lies at –6.168 eV, while the lowest unoccupied molecular orbital (LUMO) is positioned at 0.501 eV, yielding a wide bandgap that renders ADT electronically inert and prevents parasitic charge transfer or carrier trapping at the interface [17]. More importantly, we hypothesize that the rigid three-dimensional adamantane skeleton may provide a sterically bulky and chemically robust platform that could act as a physical barrier against moisture and ion migration, while also preventing excessive molecular interpenetration into the perovskite lattice, thereby helping to preserve the intrinsic optoelectronic properties of the absorber [8,14,15]. Unlike flexible thiol molecules that may adopt multiple conformations and undergo uncontrolled aggregation, the rigid cage-like architecture of ADT ensures a well-defined and reproducible interfacial arrangement, which is crucial for achieving consistent energy-level modulation and stable passivation effects [8,17]. Together, the electron-rich –SH group and rigid adamantane skeleton enable ADT to act as a dual-action modifier that provides chemical modulation and physical encapsulation, simultaneously enhancing device efficiency and stability [10,12,13].

### 2.2. Structural Characterization

To investigate the morphological and structural impacts of ADT treatment on the perovskite films, atomic force microscopy (AFM) and scanning electron microscopy (SEM) were employed [18,19,20]. Comparative analysis reveals that the ADT-treated surface exhibits negligible morphological changes compared to pristine control (Figures S1 and S2). Concurrently, X-ray diffraction (XRD) patterns confirm that the ADT modification does not alter the intrinsic crystal structure of the perovskite (Figure S3), with all characteristic diffraction peaks remaining consistent with those of the control [18,19,20]. To assess the improved moisture resistance imparted by the hydrophobic nature of the ADT molecules, water contact angle measurements were conducted (Figure S4). As anticipated, the ADT-treated films exhibit a substantially increased contact angle relative to the untreated control. A more comprehensive investigation regarding moisture-related stability will be presented in the subsequent device performance section [9,19,20]. To further elucidate the surface chemical states and interaction mechanisms induced by ADT treatment, high-resolution X-ray photoelectron spectroscopy (XPS) measurements were conducted on the Pb 4f and N 1s core levels [21]. As depicted in Figure 2a, notable shifts toward higher binding energies are observed for the ADT-treated film compared to pristine control. Specifically, both Pb 4*f*_5/2_ and Pb 4*f*_7/2_ doublet peaks exhibit a discernible positive shift of approximately 1 eV, accompanied by a concurrent distinct shift in the N 1*s* spectrum. There is also a positive shift observed in the I 3*d* spectrum (Figure S5), paralleling the shifts in Pb 4*f* and N 1*s*. These pronounced shifts clearly indicate a decrease in the electron density around both Pb, I and N atoms within the treated perovskite, implying strong interfacial chemical interactions [21,22,23]. This electronic modification can be plausibly attributed to the effective modulation of undercoordinated Pb^2+^ defects via coordination with the electron-rich functional groups (e.g., S) of the ADT molecules. Meanwhile, the shift observed in the N 1*s* peak further suggests that hydrogen bonding or electrostatic interactions may simultaneously occur between ADT and the organic cations [21,22,23,24,25]. We also performed S 2*p* XPS measurements. The ADT-treated film shows a slight enhancement in the S 2*p* region (160–164 eV) compared to the control (Figure S6), consistent with S–Pb coordination. However, due to the sub-monolayer ADT coverage, the signal remains weak and near the detection limit of lab-based XPS. To further corroborate the surface electronic modifications revealed by XPS, we performed ultraviolet photoelectron spectroscopy (UPS) measurements to examine the energy level alignment [26]. As shown in Figure 2b, the secondary electron cutoff edge shifts from 16.77 eV for the control to 16.42 eV for the ADT-treated film, corresponding to a pronounced increase in the work function from 4.45 eV to 4.80 eV. Meanwhile, the valence band maximum (VBM) exhibits a slight upshift from 1.90 eV to 1.76 eV relative to the Fermi level. The resulting energy level diagram (right panel) clearly illustrates that the substantial increase in work function, driven by the surface dipole effect, raises the vacuum level and deepens the ionization potential [19,26,27]. This energy-level realignment, which is consistent with the interfacial dipole induced by ADT, is highly beneficial for interface engineering. The enlarged work function not only strengthens the surface oxidation resistance but also promotes more favorable band alignment at the charge transport layer interface [19,27]. Consequently, it effectively suppresses interfacial charge recombination (as depicted by the suppressed electron injection) and facilitates hole extraction [19,26,27]. The microscopic surface electrical properties were characterized by Kelvin probe force microscopy (KPFM) to elucidate interfacial charge transport behaviors (Figure 2c). The control film exhibits an average surface potential of approximately 1.38 V, which notably rises to 1.63 V following ADT treatment, accompanied by a substantial upward shift in the entire potential scale. This pronounced elevation in surface potential directly corroborates the surface dipole modification induced by the ADT molecules, aligning well with the increased work function observed in our UPS measurements. Such optimized interfacial energetics are ultimately conducive to minimizing potential barriers and facilitating more efficient charge extraction at the adjacent transport layer interface [19,27,28]. In summary, the synergistic XPS, UPS, and KPFM analyses confirm that ADT treatment chemically passivates surface defects via strong electronic interactions [19,20,27]. This induces a favorable surface dipole and lowering interfacial barriers, and suppressing charge recombination [19,27].

### 2.3. Optoelectronic Characterization

To elucidate the influence of ADT modification on the optical properties of the perovskite films, UV–Vis absorption spectroscopy was employed. Notably, the ADT-treated films exhibit an absorption profile similar to that of the pristine control across the entire spectral range (Figure S7), indicating that the ADT interfacial layer does not substantially alter the intrinsic light-harvesting capability of the perovskite absorber [18]. To directly reveal how ADT modulates the early-stage carrier dynamics, we examined the transient absorption (TA) spectra of the control and treated films (Figure 3a) [29]. Two prominent spectral differences highlight the modulation efficacy. First, the ADT-treated sample exhibits a substantially deeper ground-state bleaching (GSB) dip centered at ~750 nm at early delay times (< 50 ps). Notably, the control film exhibits a higher absorbance than the ADT-treated film at the pump wavelength (380 nm; Figure 3a). Thus, under the same incident pump fluence, a higher initial photoexcited carrier density would be expected for the control film. Nevertheless, the ADT-treated film exhibits the stronger early-time GSB, indicating that the enhanced GSB cannot be attributed to a higher initial excitation density. Since the GSB amplitude is closely associated with the population of free carriers at the band edge, this enhancement strongly supports the interpretation that ADT treatment effectively suppresses the ultrafast non-radiative capture of nascent free electrons by deep trap states [30,31,32]. Second, a striking contrast is observed in the high-energy region (600–700 nm). The control film exhibits undulatory spectral features at early times (e.g., 0.5 ps), which may originate from multiple ultrafast processes, including transient spectral shifts and broadening, hot-carrier relaxation, overlapping excited-state responses, and potentially coherent lattice dynamics [30,31,32,33,34]. Conversely, the ADT-treated film exhibits a smooth and featureless baseline in this same spectral region, indicating that the chemical modulation substantially suppresses these early-time high-energy spectral modulations [30,31,32,33,34,35]. Because the present TA measurements do not uniquely distinguish these contributions, we refrain from assigning these spectral features exclusively to coherent phonon oscillations. Together, the enhanced GSB intensity and the eliminated high-energy spectral undulations support reduced ultrafast carrier loss and modified early-stage carrier relaxation following ADT treatment [30,35]. The suppression of non-radiative recombination and the prolonged carrier lifetimes were further corroborated by steady-state and time-resolved photoluminescence (PL/TRPL) measurements. As displayed in Figure 3b, the ADT-treated film exhibits a substantially enhanced PL intensity compared to the control, without any observable shift in the emission peak. This pronounced PL enhancement directly indicates that the chemical modulation effectively reduces the density of surface trap states, thereby suppressing non-radiative recombination pathways [29]. To further examine the carrier recombination dynamics, TRPL decay profiles of the control and ADT-treated films were measured (Figure 3b). The ADT-treated film exhibits a slightly slower PL decay than the control film over a substantial portion of the measured temporal window. This qualitative trend is consistent with the suppression of non-radiative recombination inferred from the enhanced steady-state PL intensity and PLQY after ADT treatment [29,36]. To gain deeper insights into the intrinsic charge transport properties of the passivated films, we performed Optical fiber coupling THz-TDS system [33]. Figure 3c shows the transmitted terahertz waveforms for the control and ADT-treated films. While the general waveform profile remains unchanged, indicating that the bulk crystal structure is preserved after ADT treatment, a substantial enhancement in the amplitude of both the positive and negative peaks is clearly observed. Specifically, the transmitted terahertz peak-to-peak amplitude is markedly increased in the treated sample compared to the pristine control. Since the terahertz transmission amplitude is directly proportional to the complex photoconductivity, this pronounced increase demonstrates that ADT modulation significantly boosts the free-carrier mobility [33,37,38,39]. THz-TDS analysis reveals that ADT-treated films exhibit a prolonged scattering time (50 ± 3 fs) compared to the control (32 ± 2 fs), along with a ~57% enhancement in mobility (105 ± 6 vs. 67 ± 4 cm^2^/(V·s)) (The detailed calculation process can be found in the supporting information), indicating suppressed carrier scattering and more efficient charge transport [33,37,38,39]. This improvement is likely attributed to the reduction in surface and grain-boundary scattering resulting from the effective chemical passivation of trap states [20]. Avoiding optical pumping eliminates possible complications from non-equilibrium carrier distributions, hot-carrier effects, and recombination dynamics. The mobility extracted by the conventional THz-TDS without optical pumping therefore reflects the intrinsic steady-state transport properties more directly. In our material, the equilibrium carrier density is high enough to provide a strong THz signal and a clear Drude roll-off within the measured spectral window. Therefore, conventional THz-TDS already allows reliable extraction of *τ* and hence the absolute mobility. Consequently, these OPTP results corroborate enhanced PL and prolonged lifetimes, further confirming that the ADT treatment not only reduces non-radiative recombination but also significantly facilitates efficient charge transport within the perovskite film [33,35]. Overall, the optical dynamics reveal that ADT modulation suppresses early-stage trap-mediated non-radiative recombination, as evidenced by enhanced PL and prolonged TRPL lifetimes. Furthermore, the improved carrier mobility confirmed by THz-TDS signifies that this modulation not only reduces recombination but also unlocks efficient charge transport pathways [29,31,32,33,34,39]. To quantitatively evaluate the suppression of non-radiative recombination and its contribution to the device performance, we conducted absolute photoluminescence quantum yield (PLQY) measurements, from which the quasi-Fermi level splitting (QFLS) was subsequently derived [40]. As presented in Figure S8, the control film exhibits a PLQY of 4.2%, which is nearly doubled to 8.2% upon ADT treatment. This remarkable enhancement directly translates into an increased thermodynamic voltage limit, with the QFLS rising from 1.21 eV for the control to 1.22 eV for the treated film (Figure S8). This improvement in QFLS is effectively translated into a corresponding enhancement in the device’s open-circuit voltage ($V_{\mathrm{oc}}$), which increases from 1.16 V to 1.17 V. The synchronized rise in both QFLS and $V_{\mathrm{oc}}$ confirms that the ADT interfacial layer not only effectively passivates surface trap states to reduce non-radiative recombination within the perovskite but also maintains excellent energy-level alignment to ensure efficient charge extraction [40]. Consequently, the voltage loss between the thermodynamic limit (QFLS) and the actual device output ($V_{\mathrm{oc}}$) remains consistently minimal, validating that the improved intrinsic photophysics are successfully translated into macroscopic photovoltaic performance [30,33,40].

### 2.4. Photovoltaic Characterization

To evaluate the practical photovoltaic performance of the ADT passivation strategy, we fabricated p–i–n planar devices fabricated in ambient air with the configuration of ITO/Me-4PACz/perovskite/ADT/PCBM/BCP/Ag, as schematically illustrated in Figure 4a, in this device architecture, Me-4PACz serves as the hole transport layer (HTL), while PCBM and BCP function as the electron transport layer (ETL). Figure 4b displays the representative current density–voltage (J–V) curves of the best-performing control and ADT-treated devices measured under standard AM 1.5G illumination. The champion ADT-treated device achieves a remarkable power conversion efficiency (PCE) of 23.9% with an open-circuit voltage ($V_{\mathrm{oc}}$) of 1.165 V, a short-circuit current density ($J_{\mathrm{sc}}$) of 24.46 mA cm^−2^, and a fill factor (FF) of 83.4%. This represents a marked improvement over the best control device, which yielded a PCE of 23.5% (with a $V_{\mathrm{oc}}$ of 1.157 V, $J_{\mathrm{sc}}$ of 24.32 mA cm^−2^, and FF of 83.2%). The enhanced short-circuit current was further corroborated by external quantum efficiency (EQE) measurements (Figure S9). Compared to the control, the ADT-treated device exhibits a consistently higher photo response across the entire visible spectrum, confirming the reliability of the improved charge collection and the effectiveness of ADT passivation [20,41]. The statistical distributions of the photovoltaic parameters for 10 individual devices are summarized in the box charts in Figure 4c. The treated devices exhibit a notable and consistent enhancement in all key parameters, particularly in $V_{\mathrm{oc}}$ and $J_{\mathrm{sc}}$, which agrees well with the suppressed non-radiative recombination and improved carrier mobility demonstrated by the optical characterizations. SCLC analysis reveals a prominent reduction in the trap-filled limit voltage ($V_{\mathrm{TFL}}$) upon ADT treatment (Figure S10), quantitatively confirming the effective passivation of trap states [41,42]. We also tested a lower ADT concentration (0.5 mg/mL). The champion PCE was slightly improved to 21.71%, compared with 21.33% for the freshly prepared control (Figure S11). However, the overall statistical data show that Voc, Jsc, FF, and PCE remained essentially comparable between the two groups (Figure S12), indicating that 0.5 mg/mL is below the effective concentration threshold for ADT passivation. To further investigate the passivation functionality of ADT, we extended our study to conventional n-i-p devices with the architecture ITO/SnO_2_/Perovskite/ADT/Spiro-OMeTAD/Ag (Figures S13–S15). All solution-processing steps were performed in ambient air, consistent with the fabrication protocol for the inverted devices. The ADT-treated champion device achieved a PCE of 23.91%, substantially higher than that of the control device (20.86%). Notably, a significant improvement in fill factor (FF) was observed (Figure S14), suggesting that ADT optimizes the interfacial energy alignment and facilitates hole extraction toward the Spiro-OMeTAD layer in the n-i-p configuration [41,42]. In contrast, the inverted (p-i-n) devices showed only a modest PCE enhancement around 0.4%, which we attribute to the unfavorable energy-level alignment at the perovskite/PCBM interface. Despite this energetic mismatch, ADT still effectively passivates surface defects and suppresses non-radiative recombination at the interface, as evidenced by the enhanced PL and prolonged carrier lifetimes. Furthermore, the unencapsulated device stability was assessed by tracking the maximum power point (MPP) under continuous one-sun illumination. As shown in Figure 4d, the ADT-treated device retains over 95% of its initial PCE after 400 h of continuous operation, while the control device retains approximately 90% of its initial PCE. This remarkable operational stability underlines the robustness of the ADT interfacial passivation against light-induced degradation, firmly proving that ADT treatment is a highly effective and reliable strategy for boosting both efficiency and long-term durability of perovskite solar cells.

## 3. Discussion

The ADT molecule features a rigid cage-like backbone with a high rotational energy barrier, immobilizing its molecular configuration and keeping functional groups anchored to surface defect sites during prolonged operation. The outward orientation of the adamantane skeleton and the interfacial dipole were confirmed by UPS, KPFM, contact angle measurements and DFT calculation. UPS shows a work function shift and KPFM an increase in surface potential, both indicating a net dipole. The increased contact angle further confirms the hydrophobic adamantane cage points outward while –SH anchors to Pb^2+^. DFT charge density calculations further reveal that the –SH group has the strongest binding affinity to the perovskite surface, supporting the proposed anchoring geometry. The hydrophobic ADT skeleton also impedes water infiltration into the perovskite lattice, preserving coordination bonds with undercoordinated Pb^2+^ defects and preventing the passivation effect from collapsing over time. This chemical robustness likely underlies the exceptional long-term MPP stability observed in our unencapsulated devices. Future studies could incorporate time-resolved in situ GI-XRD to monitor real-time crystallographic evolution under high humidity, confirming the delayed formation of the hydrated PbI_2_ phase [43,44]. We believe this rigid-molecule-based passivation strategy is robust and universal for fabricating high-performance perovskite devices with superior ambient tolerance and operational durability.

## 4. Materials and Methods

### 4.1. Materials

FAI (99.99%, trace elements basis), methylammonium iodide (99.99%, trace elements basis) and methylammonium chloride (99.99%, trace elements basis) were purchased from Lumtec. PbI_2_ (99.99%, trace metals basis) was purchased from TCI (Tokyo, Japan). Self-assembly molecules of [4-(3,6-dimethyl-9H-carbazol-9-yl)butyl]phosphonic acid (Me4PACz) (>99%) were purchased from Lumtec (New Taipei City, Taiwan, China). Caesium iodide (99.999%) was purchased from Sigma Aldrich (St. Louis, MO, USA). 6,6-phenyl-C_61_-butyric acid methyl ester (PCBM) (≥99.99%) and 2,2′,7,7′-tetrakis[N,N-di(4-methoxyphenyl)-amino]-9,9′-spirobifluorene (spiro-OMeTAD) were obtained from Greatcell Solar Materials (Queanbeyan, NSW, Australia). Bathocuproine (BCP) (99.8%) was acquired from Ossila (Sheffield, UK). Ag shots (2–3 mm, 99.999%) and 15% SnO_2_ nanoparticle solution were bought from Alfa Aesar (Ward Hill, MA, USA). N, N-dimethylformamide (DMF), isopropyl alcohol (IPA), diethyl ether (DEE), 75 weight % in IPA absolute ethanol and anhydrous chlorobenzene, 4-tert-butyl pyridine (tbp), lithium bistrifluorosulfonyl imide (Li-TFSI), acetylacetone, phenethylamine and titanium diisopropoxide bis(acetylacetonate) were acquired from Sigma-Aldrich (St. Louis, MO, USA). All the chemicals were used as received without further purification. Conductive glass, ITO (10 ohms/sq), was purchased from Nippon Sheet Glass (Tokyo, Japan), and 1-adamantanethiol (ADT) (≥99.9%) was purchased from Adamas Reagent, Ltd (Shanghai, China).

### 4.2. Preparation of Perovskite Precursor Solution

The 1.50 M perovskite precursor solution was prepared by dissolving PbI_2_, CsI, FAI, MABr, and PbBr_2_ in a DMF:DMSO (4:1, *v*/*v*) mixed solvent to obtain Cs_0.05_(FA_0.98_MA_0.02_)_0.95_Pb(I_0.98_Br_0.02_)_3_. To optimize device performance, 5 mol% excess PbI_2_ and 15 mol% MACl were added to the precursor solution.

### 4.3. Perovskite Solar Cell Fabrication

Inverted PSCs were fabricated with a glass/ITO/Me4PACz/perovskite/PCBM/BCP/Ag architecture. The glass/ITO substrates were sequentially washed with soapy water, deionized water, acetone, and isopropanol under sonication for 30 min each. After drying with N_2_, the cleaned substrates were treated with O_2_ plasma for 15 min. Me4PACz in ethanol (1 mg/mL) was spin-coated onto the treated substrates at 3000 rpm for 30 s and annealed at 100 °C for 10 min. The perovskite precursor solution (60 µL) was then spin-coated at 1000 rpm for 10 s and 4000 rpm for 30 s, with 200 µL of CB dripped onto the center 15 s before the end of the spin-coating. The films were immediately annealed at 100 °C for 60 min. ADT in CB (1 mg/mL) was spin-coated onto the perovskite surface at 3000 rpm for 30 s. Finally, PCBM (25 nm), BCP (10 nm), and Ag (100 nm) were sequentially deposited by thermal evaporation under high vacuum (<1 × 10^−5^ Pa).

The n-i-p perovskite solar cells were fabricated with a device architecture of ITO/SnO_2_/Perovskite/Spiro-OMeTAD/Ag. Patterned ITO substrates were sequentially cleaned with detergent, deionized water, acetone, and isopropanol under ultrasonication, followed by UV–ozone treatment for 15 min. A diluted SnO_2_ colloidal solution was spin-coated onto the cleaned substrates at 3000 rpm for 30 s and subsequently annealed at 150 °C for 30 min to form the electron transport layer. The perovskite precursor solution was prepared by dissolving FAI, PbI_2_, PbBr_2_, MABr, and CsI in a mixed solvent of DMF and DMSO. The precursor solution was spin-coated using a two-step program (1000 rpm for 10 s and 5000 rpm for 30 s). During the second step, chlorobenzene was dynamically dripped onto the rotating substrate as an antisolvent to induce rapid crystallization. The obtained films were annealed at 100 °C for 20 min to form a compact perovskite absorber layer. For surface passivation, 1 mg/mL ADT solution in CB was spin-coated onto the perovskite films and annealed at 100 °C for 5 min. Subsequently, the hole transport layer was deposited by spin-coating a Spiro-OMeTAD solution (72.3 mg mL^−1^ in chlorobenzene containing Li-TFSI and tBP additives) at 3000 rpm for 30 s. The devices were then stored overnight in dry air to promote Spiro-OMeTAD oxidation. Finally, an 80 nm Ag electrode was thermally evaporated under high vacuum.

### 4.4. X-Ray Photoelectron Spectroscopy (AXIS SUPRA)

XPS was performed using an Al Kα radiation source, with binding energies calibrated against the C 1 s peak at 284.8 eV.

### 4.5. Ultraviolet Photoelectron Spectroscopy (UPS)

(UPS) measurements were performed using a Thermo Scientific ESCALAB 250Xi system (Thermo Fisher Scientific, Waltham, MA, USA) equipped with a He I (21.22 eV) ultraviolet excitation source under ultra-high vacuum conditions (pressure < 1 × 10^−9^ mbar). The secondary electron cutoff was measured with a −5.0 V bias applied to the sample to enhance detection accuracy. The Fermi level was calibrated using a clean Au reference prior to measurement. The work function (Φ) of the samples was determined from the secondary electron cutoff region, while the valence band maximum (VBM) position was extracted from the low binding energy region of the spectra. All measurements were carried out at room temperature.

### 4.6. Photoluminescence Spectroscopy (PL)

PL spectra were recorded using a Fluorolog-322 spectrometer (Horiba Jobin Yvon iHR320, Edison, NJ, USA) equipped with a CCD detector and a Xenon lamp (excitation at 350 nm), with a slit width of 5 nm.

### 4.7. Time-Resolved Photoluminescence (TRPL)

TRPL measurements were performed with time-correlated single photon counting (TCSPC) using a HORIBA Fluorolog-QM spectrofluorometer (HORIBA Scientific, Piscataway, NJ, USA). The excitation was provided by a 407 nm DeltaDiode (HORIBA Scientific, Piscataway, NJ, USA) picosecond laser diode (50 ps) at 100 MHz repetition rate. The samples were measured in air.

### 4.8. Transient Absorption (TA) Spectroscopy

TA system is home-built based on a Coherent Astrella femtosecond laser, which delivers 35 fs pulses centered at 800 nm with a repetition rate of 1 kHz and an average power of 7 W. Forty percent of the laser output is used to pump an optical parametric amplifier (TOPAS Twins, Light Conversion, Vilnius, Lithuania) to generate the pump and probe beams. The pump pulse was generated by a BBO crystal for the second harmonic generation (380 nm) from TOPAS Twins which tuned to 760 nm. The pump beam passes through an optical chopper operating at 500 Hz and a motorized variable neutral density filter before being collinearly focused onto the sample, overlapping spatially with the probe beam. The probe output from the TOPAS Twins is tuned to 1300 nm and then directed through a mechanical delay line. This beam is used to generate a supercontinuum broadband white-light in a rapidly moving 5-mm-thick CaF_2_ window. The probe light is then focused onto the sample using an off-axis parabolic mirror, providing a maximum delay time of 8 ns. Both pump and probe pulses were temporally and spatially overlapping on the sample. After transmission through the sample, the probe pulses and a reference probe were sent to a prism spectrometer with a CCD array. The TA signal is derived from the differential absorption, calculated as the intensity difference between the pump-on and pump-off conditions.

### 4.9. Kelvin Probe Force Microscopy (KPFM)

KPFM measurements were conducted using a Jupiter XR atomic force microscope (Oxford Instruments, Abingdon, UK) operating in amplitude-modulated KPFM mode under ambient conditions. Conductive Pt/Ir-coated silicon probes with a nominal resonance frequency of approximately 75 kHz were employed. Surface topography and contact potential difference (CPD) maps were simultaneously acquired in a two-pass scanning mode. The lift height was set to 30 nm during CPD measurements. The obtained surface potential distributions were analyzed to evaluate the effect of surface treatment on the electronic properties and charge distribution of the perovskite films.

### 4.10. Current Density–Voltage (J–V) Characteristics

Current density–voltage (J–V) characteristics of the perovskite solar cells were measured using a solar simulator (Enlitech SS-F5-3A, Kaohsiung, Taiwan, China) equipped with a class AAA LED light source providing AM 1.5G spectrum (100 mW cm^−2^). The light intensity was calibrated using a certified silicon reference cell prior to each measurement.

### 4.11. Maximum Power Point Tracking (MPPT)

MPPT measurements were conducted to evaluate the stabilized output performance of the devices under standard AM 1.5G illumination (100 mW cm^−2^). The tests were carried out in ambient air using a high-stability LED solar simulator (Wavelabs Sinus-70, Leipzig, Germany) coupled with a Keithley 2450 source meter. The light intensity was calibrated using a certified silicon reference cell prior to each measurement.

### 4.12. Terahertz Time-Domain Spectroscopy (THz-TDS)

Terahertz time-domain spectroscopy measurements were performed using an optical fiber coupling type THz-TDS system (QT-TO1000, Qingdao Qingyuan Fengda Terahertz Technology Co., Ltd., Qingdao, China). This system employs photoconductive antennas for terahertz wave generation and electro-optic sampling for coherent detection, enabling the direct acquisition of the time-domain electric field waveforms of the terahertz pulses. Measurements were conducted at room temperature under low-humidity conditions with a relative humidity of <10%. The system provides a usable spectral bandwidth of approximately 0.1–5 THz and a spectral dynamic range exceeding 80 dB The time delay of the probe pulses was precisely controlled using an optical delay line, achieving a time-domain scanning length of >300 ps and a frequency resolution of <5 GHz. During the measurements, the terahertz waves traversed the samples in either transmission or reflection modes. The acquired time-domain signals were subsequently transformed into the frequency domain using fast Fourier transform (FFT). By comparing the amplitude and phase differences between the sample signals and the reference signals (obtained without a sample), the optical parameters of the samples in the terahertz regime—including the refractive index, absorption coefficient, and complex permittivity—were successfully derived.

### 4.13. Photoluminescence Quantum Yield (PLQY) Measurements

Absolute photoluminescence quantum yield (PLQY) spectra were recorded using an Edinburgh Instruments FLS1000 fluorescence spectrometer (Edinburgh Instruments, Livingston, UK) equipped with an integrating sphere. The perovskite films deposited on quartz or glass substrates were placed at the center of the integrating sphere. The excitation wavelength was set at 532 nm using a monochromatic xenon lamp. The laser intensity was set to 1.0 sun, with a laser spot size of 0.10 cm^2^ and a sub cell area of 1.000 cm^2^, giving an EQE of 0.90 at the laser wavelength.

## 5. Conclusions

In summary, we have demonstrated a highly effective surface passivation strategy for p–i–n perovskite solar cells fabricated in ambient air by employing a rigid cage-like small molecule (ADT) as an interfacial modifier, with all solution-processing steps performed in ambient air (20–25 °C, 40–60% RH). A comprehensive suite of spectroscopic and optoelectronic characterizations reveals the underlying working mechanism: the electron-rich functional groups of the ADT molecule strongly coordinate with undercoordinated Pb^2+^ defects at the perovskite surface. This chemical interaction induces a favorable surface dipole and a notable increase in work function, as corroborated by XPS and UPS analyses, thereby optimizing the interfacial energy alignment and homogenizing the surface potential landscape. Consequently, the ADT treatment effectively suppresses non-radiative recombination, substantially enhances free-carrier mobility, and prolongs carrier lifetimes, as directly evidenced by TA, steady-state/time-resolved PL, and THz-TDS measurements.

Benefiting from these synergistic improvements, the ADT-modified devices fabricated in ambient air achieve a champion power conversion efficiency of 23.9%, along with significantly enhanced operational stability, retaining over 95% of their initial efficiency after 400 h of continuous maximum power point tracking under one-sun illumination. Crucially, the rigid molecular skeleton of ADT not only provides robust chemical stability against thermal and photo-induced stresses but also acts as an efficient hydrophobic barrier against moisture degradation, which is consistent with increased surface hydrophobicity. This work highlights the critical role of molecular rigidity in designing effective passivation interlayers and presents a universal, ambient-air-compatible strategy for fabricating high-efficiency, long-term durable perovskite solar cells.

## Figures and Tables

**Figure 1:**
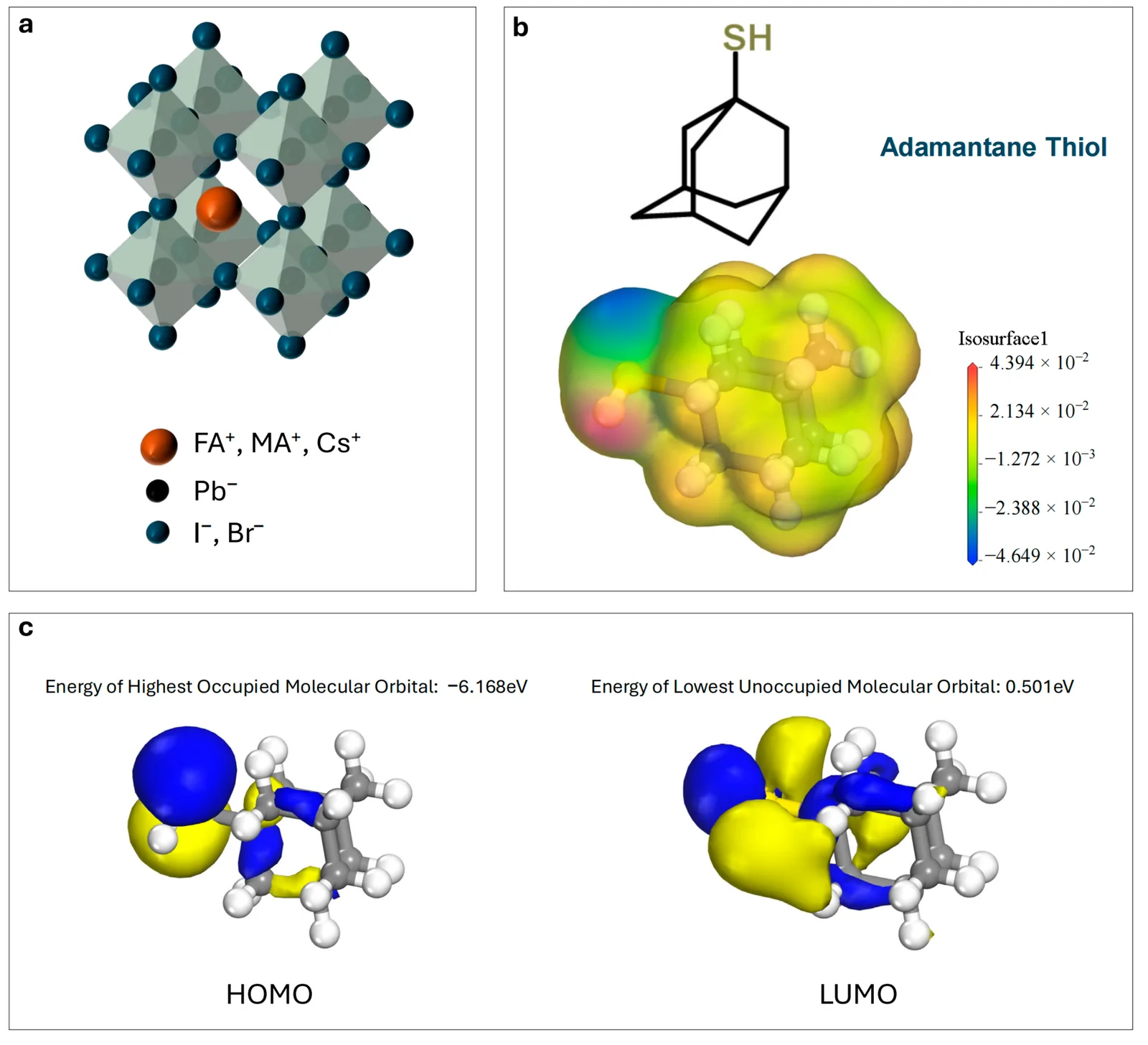
(**a**) Schematic illustration of the perovskite ABX_3_ crystal structure, where A-site cations (FA^+^, MA^+^, or Cs^+^) are coordinated with Pb^2+^ cations at the B-site and halide anions (I^−^, Br^−^, or Cl^−^) at the X-site in an octahedral geometry. (**b**) Chemical structure of 1-adamantanethiol (ADT) and electrostatic potential (ESP) map of ADT, where the negative potential (blue) is predominantly localized around the –SH group, indicating its strong electron-donating character and affinity for undercoordinated Pb^2+^ ions. (**c**) Calculated frontier molecular orbitals (HOMO and LUMO) of ADT, with energy levels of –6.168 eV and 0.501 eV, respectively, confirming its electronically inert nature that prevents undesirable charge transfer at the perovskite interface.

**Figure 2:**
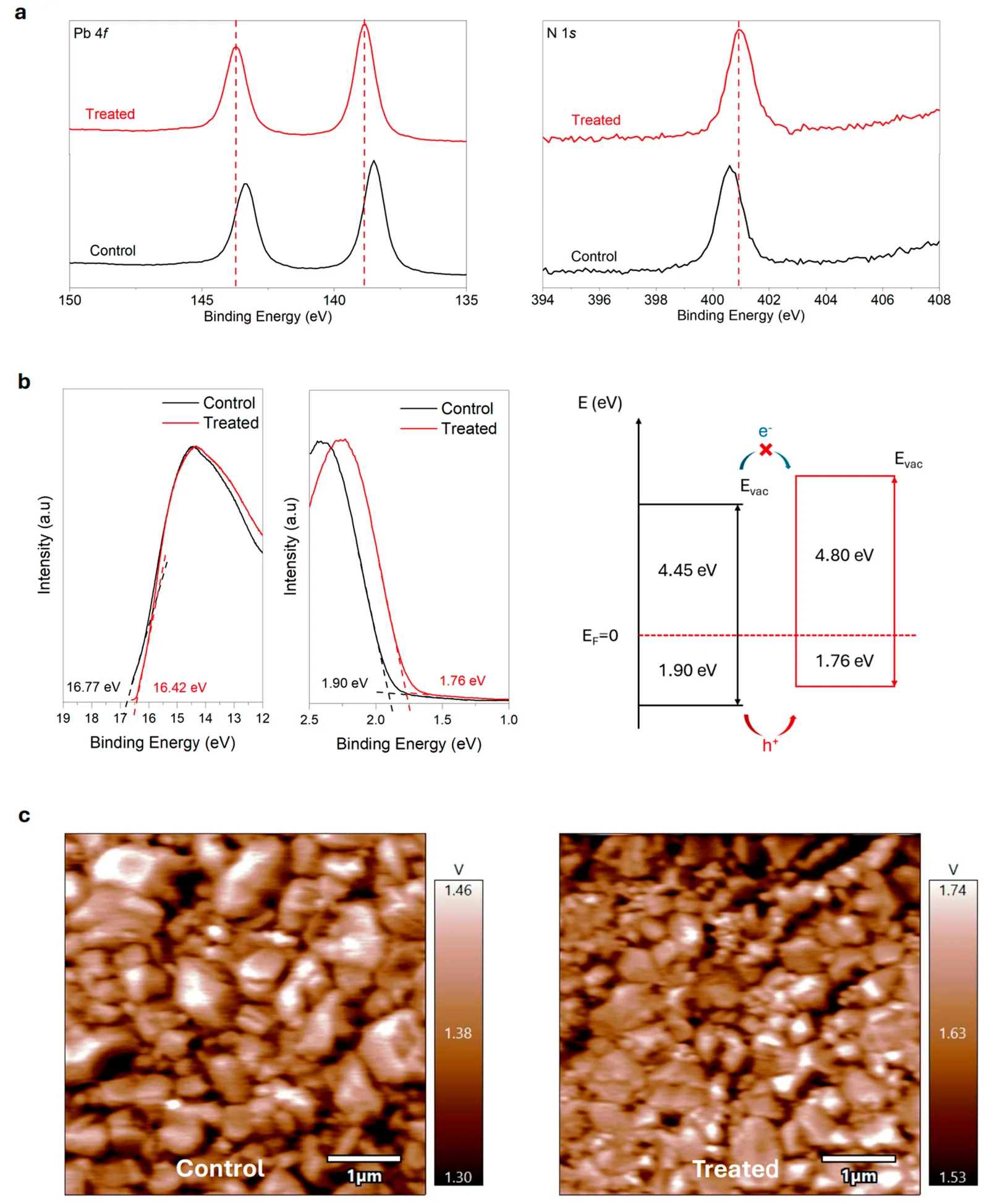
Characterization of the surface chemistry and electronic structures. (**a**) Pb 4f and N 1s XPS spectra exhibiting binding energy shifts after ADT treatment. Dashed lines indicate the target peak positions. (**b**) UPS spectra demonstrating a work function increase from 4.45 eV (control) to 4.80 eV (treated). (**c**) KPFM surface potential images (scale bars: 1 µm) showing a uniformly elevated potential for the ADT-treated film, indicating effective surface modulation and favorable energy-level alignment.

**Figure 3:**
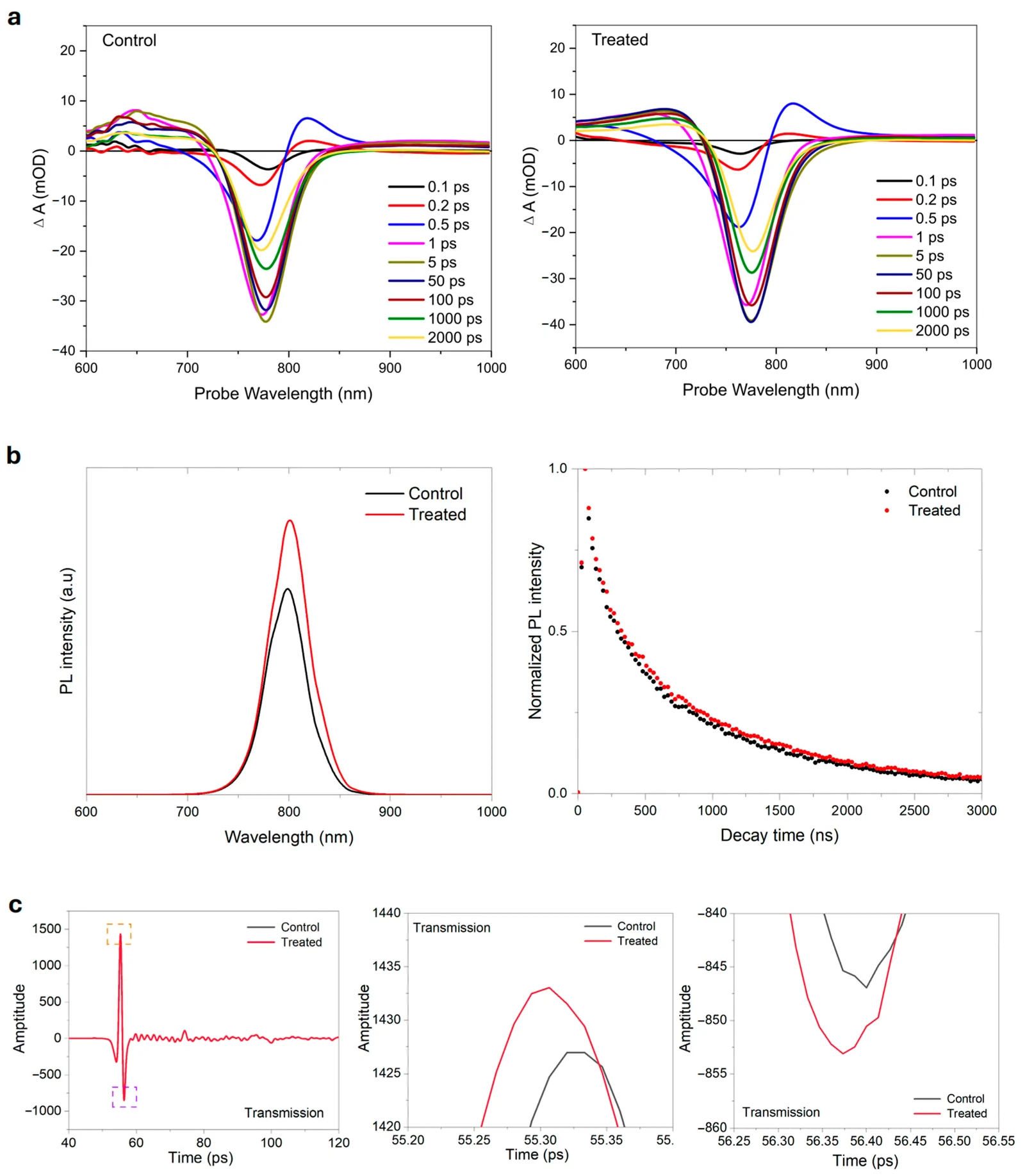
Charge carrier dynamics probed by optical spectroscopies. (**a**) TA spectra showing enhanced early-stage carrier population and reduced spectral undulations after ADT treatment. (**b**) Steady-state PL and TRPL decay curves, demonstrating enhanced radiative recombination and prolonged carrier lifetimes. (**c**) THz-TDS transmission waveforms, indicating a marked increase in free-carrier mobility. Dashed boxes mark the regions that are magnified in the next panels. Together, these results show that ADT modulation suppresses trap-mediated recombination and facilitates charge transport.

**Figure 4:**
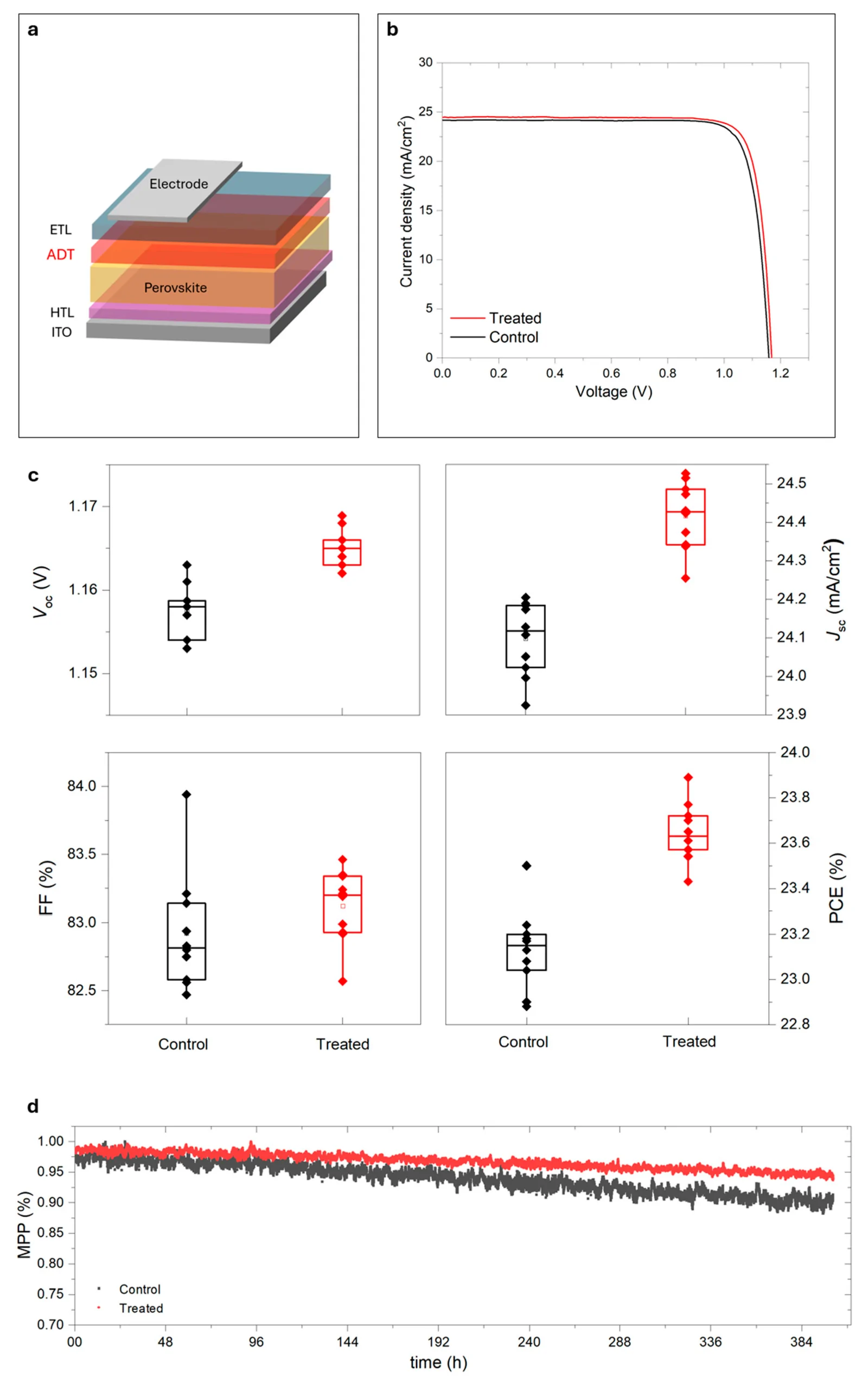
Device performance and long-term stability of p–i–n perovskite solar cells. (**a**) Device architecture (ITO/Me-4PACz/perovskite/ADT/PCBM/BCP/Ag). (**b**) J–V curves of the champion control and ADT-treated devices, showing an increased PCE from 23.5% to 23.9%. (**c**) Statistical distributions of the key photovoltaic parameters for 10 independent devices, highlighting the improved $V_{\mathrm{oc}}$, $J_{\mathrm{sc}}$, and FF (mean ± standard deviation in Table S1). (**d**) MPP tracking of unencapsulated devices under continuous illumination, demonstrating that the ADT-treated cell retains over 95% of its initial efficiency after 400 h, compared to 90% for the control.

## Data Availability

The original contributions presented in this study are included in the article. Further inquiries can be directed to the corresponding author.

## Supplementary Materials

The following supporting information can be downloaded at: https://www.mdpi.com/article/10.3390/molecules31193485/s1, Figure S1: AFM topographic images of the (a) control and (b) ADT-treated perovskite films. The treated film shows a reduced height scale (±55.0 nm) compared to the control (±80.0 nm), indicating that the ADT modification results in a smoother surface without altering the basic grain morphology; Figure S2: SEM micrographs of the (a) control and (b) ADT-treated perovskite surfaces; Figure S3: XRD patterns of the control and ADT-treated perovskite films; Figure S4: Water contact angle images of the control and ADT-treated films taken at 1 min and 3 min; Figure S5: I 3d XPS spectra of the control and ADT-treated perovskite films; Figure S6: S 2p XPS spectra of the control and ADT-treated perovskite films; Figure S7: UV–Vis absorption spectra of the control and ADT-treated films; Figure S8: (a) Absolute PLQY of the control and ADT-treated perovskite films. (b) Comparison of QFLS and device $V_{\mathrm{oc}}$; Figure S9: EQE spectra and integrated $J_{\mathrm{sc}}$ curves of the control and ADT-treated p-i-n devices; Figure S10: Dark current-voltage characteristics of electron-only devices plotted on a log-log scale for space-charge-limited current (SCLC) analysis; Figure S11:J–V curves of the champion control and 0.5mg/mL ADT-treated p-i-n perovskite solar cells measured under AM 1.5G illumination. The 0.5mg/mL ADT-treated device achieves a PCE of 21.71%, compared with 21.33% for the freshly prepared control; Figure S12: Photovoltaic parameters of control and 0.5mg/mL ADT-treated p-i-n perovskite solar cells. The 0.5mg/mL ADT-modified devices exhibit comparable open-circuit voltage (V_OC_), short-circuit current density (J_SC_), fill factor (FF) and PCE compared to the control devices; Figure S13: J–V curves of the champion control and ADT-treated n-i-p perovskite solar cells measured under AM 1.5G illumination. The ADT-modified device achieves a PCE of 23.91%, significantly outperforming the control device (20.86%); Figure S14: Photovoltaic parameters of control and ADT-treated n-i-p perovskite solar cells. The ADT-modified devices exhibit enhanced open-circuit voltage (V_OC_), short-circuit current density (J_SC_), and fill factor (FF), leading to a significantly improved PCE compared to the control devices, demonstrating the effective passivation and interfacial optimization by ADT; Figure S15: EQE spectra and integrated $J_{\mathrm{sc}}$ curves of the control and ADT-treated n-i-p devices; Table S1: The mean ± standard deviation values for all photovoltaic parameters of both control and ADT-treated n-i-p devices. Refs [45,46] are cited in the Supplementary Materials file.
