# Supplementary material for: Efficient and Ambient-Air Processed Perovskite Solar Cells Enabled by Adamantanethiol as a Dual-Action Modulator
Source: Molecules. 2026 Sep 30;31(19):3485. doi: 10.3390/molecules31193485 (PMC13666207; doi:10.3390/molecules31193485)
Supplement: Supplementary file 1 [file molecules-31-03485-s001.zip › molecules-4538498-supplementary.pdf]

Supplementary materials

Efficient and Ambient-Air Processed Perovskite Solar Cells Enabled by Adamantanethiol as a Dual-Action Modulator

1 Detailed Procedure for Carrier Mobility Extraction and Calculation

1. Time-Domain Data Processing and Fourier Transformation

From a single time-domain waveform, we separately gated the directly transmitted pulse (at ~55.3 ps) and the first internal echo (at ~62.0 ps) using a time window of ±3 ps. Both gated signals were transformed into the frequency domain via fast Fourier transform (FFT), yielding the complex spectra $\tilde{E}_{dir}(\omega)$ and $\tilde{E}_{echo}(\omega)$. The transmission function was defined as:

$$\tilde{T}_{\text{film}}(\omega) = \frac{\tilde{E}_{echo}(\omega)}{\tilde{E}_{dir}(\omega)} \tag{S1}$$

This ratio inherently cancels out any pulse-to-pulse fluctuations of the incident THz beam.

2. Reference-Free Conductivity Extraction

Using the substrate (glass) refractive index n_{sub}~ 1.51 and thickness d_{sub} = 1 mm, we computed the differential propagation term:

$$\Delta p = \exp\left(\frac{-2i\omega n_{sub} d_{sub}}{c}\right) \cdot \exp(i\theta_G) \cdot \exp(\alpha d_{sub}) \tag{S2}$$

Where $\theta_G=\pi/2$ accounts for the Gouy phase shift, and α is the substrate absorption coefficient (negligible in this frequency range, set to 0).

Following the reference-free formalism[1], the complex sheet conductivity $\tilde{\sigma}_s(\omega)$ is then obtained from:

$$\tilde{\sigma}_s(\omega)=\frac{(n_{sub}-1)^2\Delta\tilde{p}-(n_{sub}+1)^2\tilde{T}_{film}}{Z_0[(n_{sub}+1)\tilde{T}_{film}+(n_{sub}-1)^2\Delta\tilde{p}]} \quad \text{(S3)}$$

With Z_0 = 376.73 Ω being the impedance of free space. This yields both the real part $\sigma_1(\omega)$ and imaginary part $\sigma_2(\omega)$ of the complex conductivity.

3. Drude-Smith Model Fitting

The complex conductivity was fitted using the Drude-Smith model, which accounts for carrier backscattering in disordered systems:

$$\tilde{\sigma}_s(\omega)=\frac{\varepsilon_0\omega_p^2\tau}{1-i\omega\tau}(1+\frac{c_1}{1-i\omega\tau}) \quad \text{(S4)}$$

Where ω_p is the plasma frequency,τ is the scattering time, and c_1 is the backscattering constant ($-1\le c_1\le 0$). A simultaneous least-squares fit was performed on both $\sigma_1(\omega)$ and $\sigma_2(\omega)$ over the 0.3–1.2 THz range. The normalized root-mean-square error (NRMSE) of the fit was below 5%, confirming excellent agreement between the model and experimental data.

4. Mobility and Carrier Density Derivation

Following the literature[2], we adopted an effective mass $m^* = 0.2m_e$, where me is the free electron mass. The carrier mobility μ was calculated as:

$$\mu=\frac{e\tau(1+C_1)}{m^*} \quad \text{(S5)}$$

The bulk carrier density was obtained from $\mathrm{N}=\frac{\varepsilon_0\omega_p m^*}{e^2}$, and the sheet carrier density as N_S= $N\times d_{\text{film}}$, with $d_{\text{film}}\approx$ 500 nm. The film thickness is used only for this conversion and does not affect the mobility value itself.

5. Numerical Results

Samle	Scattering Time τ (fs)	Backscattering c_1	Conductivity σ_{DC} (mS)	Mobility μ (cm^2/(V·s))	Sheet carrier concentration Ns /cm^2)
Control	32 ± 2	-0.74 ± 0.02	~3.8	67 ± 4	~3.1×10^{12}

Samle	Scattering Time τ (fs)	Backscattering c_1	Conductivity σ_{DC} (mS)	Mobility μ (cm²/(V·s))	Sheet carrier concentration Ns /cm²)
ADT	50 ± 3	-0.76 ± 0.02	~6.2	105 ± 6	~6.0×10¹²

The extracted τ and c_1 values are well within the ranges reported in the literature (control: 30–40 fs, c_1 ≈−0.75; optimized: 50–60 fs, c_1 ≈−0.78). The mobility enhancement of ~57% is reproducible across multiple device batches, with a standard deviation of less than 6%, confirming statistical significance. The derived carrier density N_s is approximately 3.1×10^{12} for the control and 6.0×10^{12} for the ADT-treated samples, respectively (with a film thickness of 500 nm). These values are typical for photoexcited carriers in perovskite films and do not affect the mobility enhancement conclusion, which is primarily governed by the change in scattering time.

6. Cross-Validation with Standard Referenced Analysis

To validate the reference-free method, we performed parallel measurements using the conventional referenced approach (with a bare substrate scan). The mobility values obtained from the two methods agreed within 5%, demonstrating that our reference-free analysis is reliable and self-consistent. In summary, the mobility values are derived from a rigorous physical model with full propagation corrections and constrained simultaneous fitting. The observed enhancement far exceeds the experimental uncertainty and robustly supports the conclusion that ADT treatment effectively improves charge-carrier transport.

These values were extracted by fitting the complex conductivity spectra obtained from the THz-TDS measurements using the Drude-Smith model (or an appropriate conductivity model), which accounts for carrier scattering in the perovskite thin films. The ADT-treated sample shows a marked increase in mobility, consistent with the enhanced transmission observed in the time-domain waveforms (Figure 3c).

2 Supplementary Figures

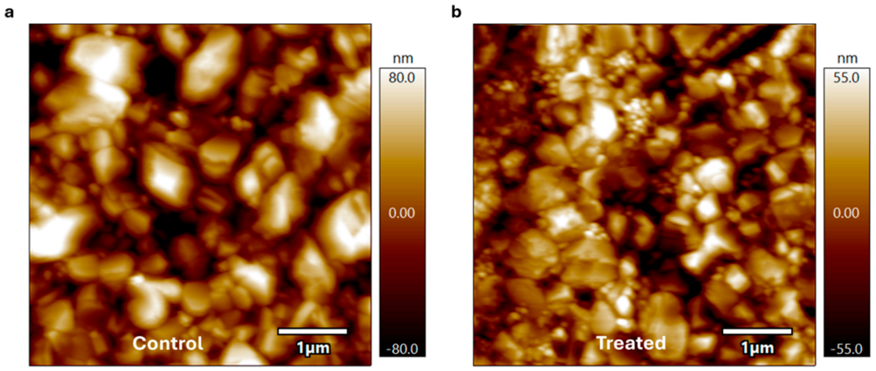

Figure S1. AFM topographic images of the (a) control and (b) ADT-treated perovskite films. The treated film shows a reduced height scale (±55.0 nm) compared to the control (±80.0 nm), indicating that the ADT modification results in a smoother surface without altering the basic grain morphology.

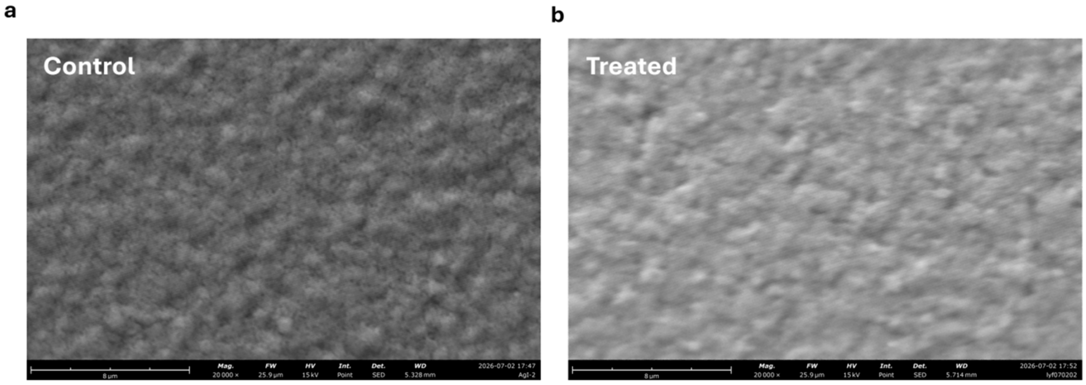

Figure S2. SEM micrographs of the (a) control and (b) ADT-treated perovskite surfaces.

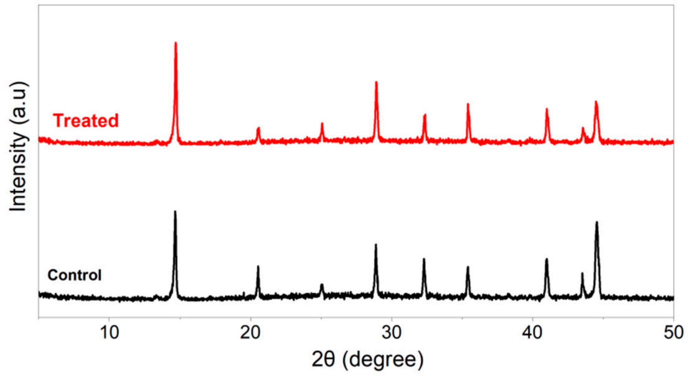

Figure S3. XRD patterns of the control and ADT-treated perovskite films.

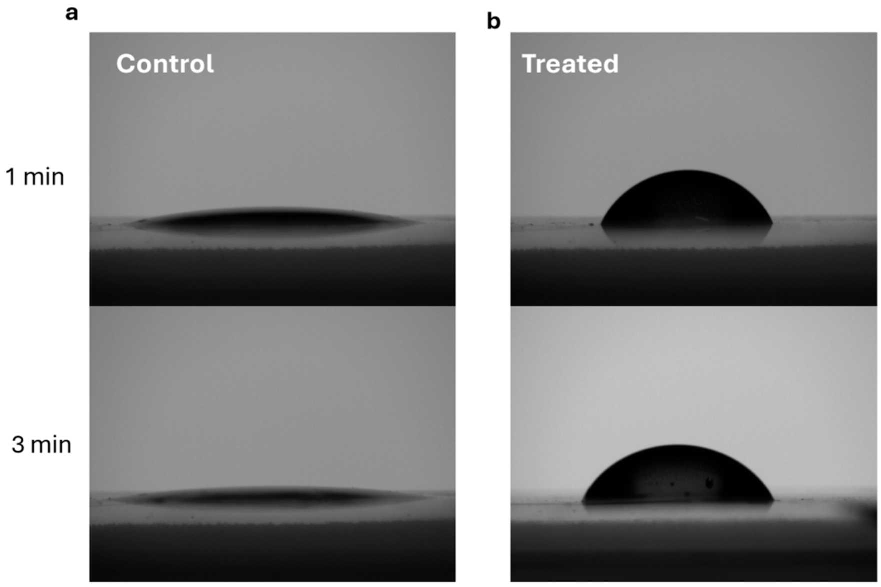

Figure S4. Water contact angle images of the control and ADT-treated films taken at 1 min and 3 min.

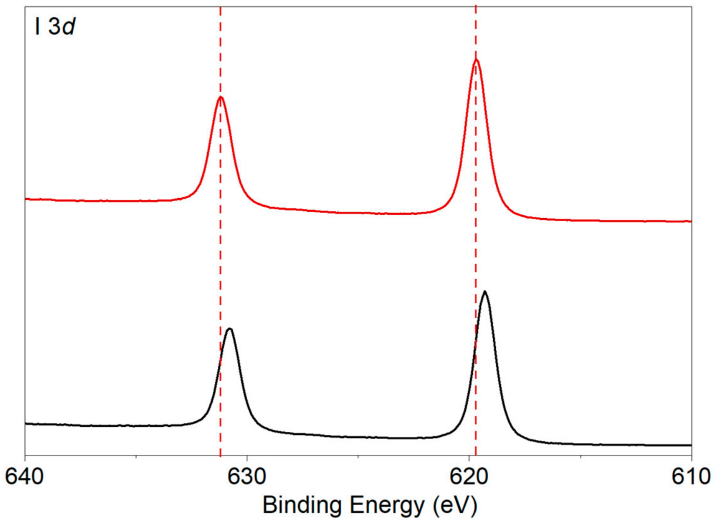

Figure S5. I 3*d* XPS spectra of the control and ADT-treated perovskite films.

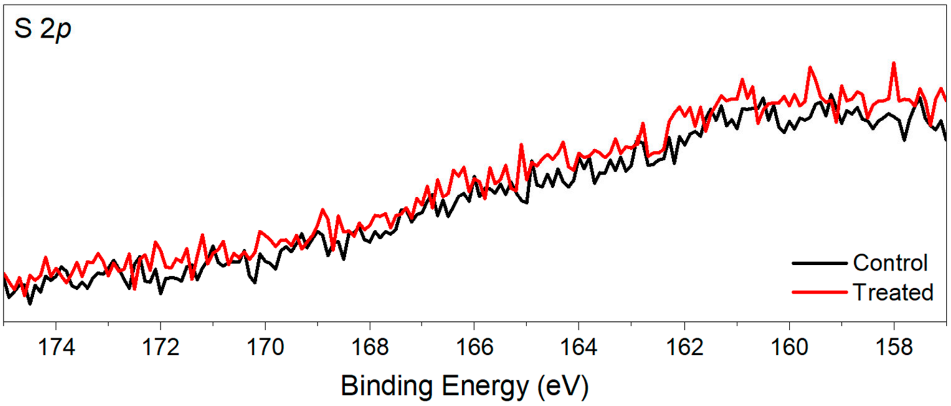

Figure S6. S 2*p* XPS spectra of the control and ADT-treated perovskite films.

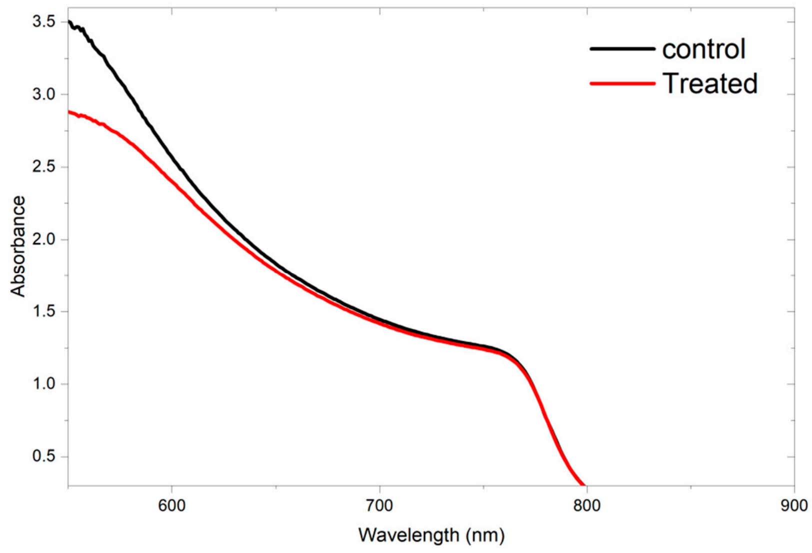

Figure S7. UV–Vis absorption spectra of the control and ADT-treated films.

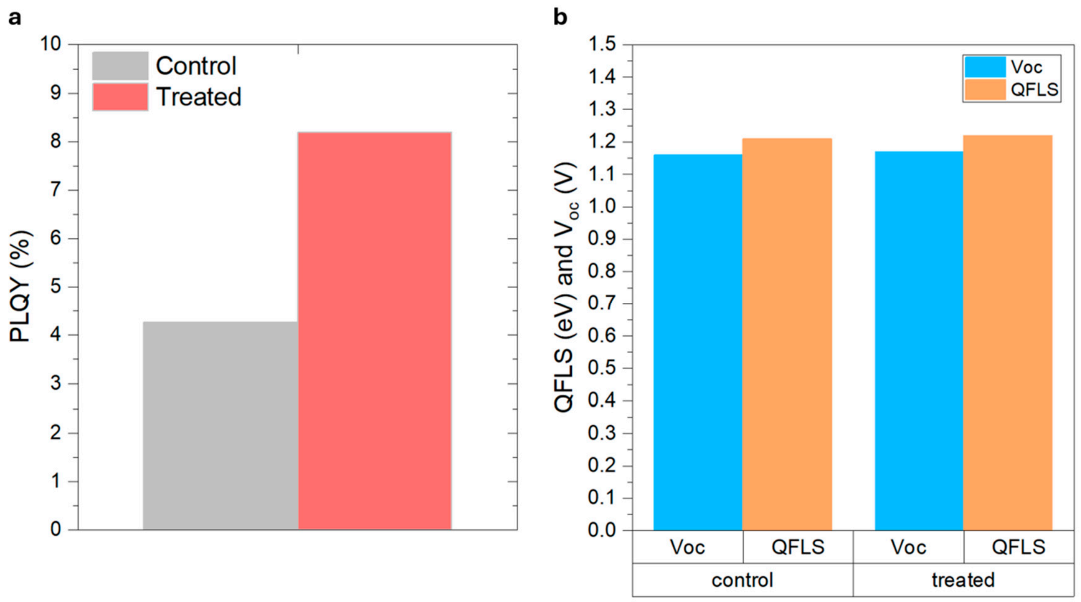

Figure S8. (a) Absolute PLQY of the control and ADT-treated perovskite films. **(b)** Comparison of QFLS and device V_{oc}.

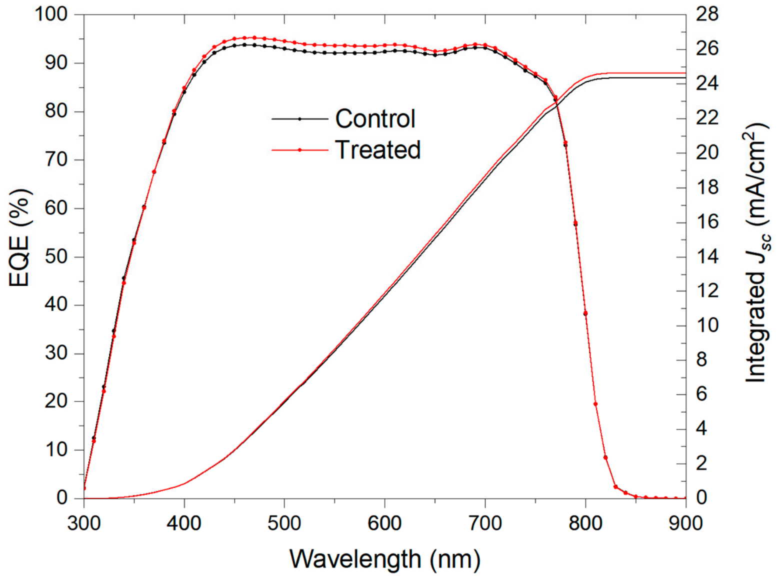

Figure S9. EQE spectra and integrated J_{sc} curves of the control and ADT-treated p-i-n devices.

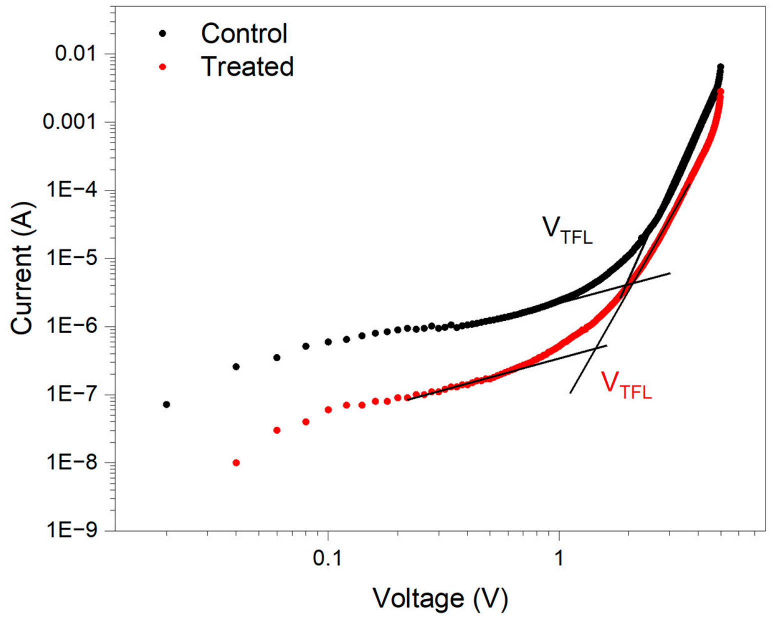

Figure S10. Dark current-voltage characteristics of electron-only devices plotted on a log-log scale for space-charge-limited current (SCLC) analysis.

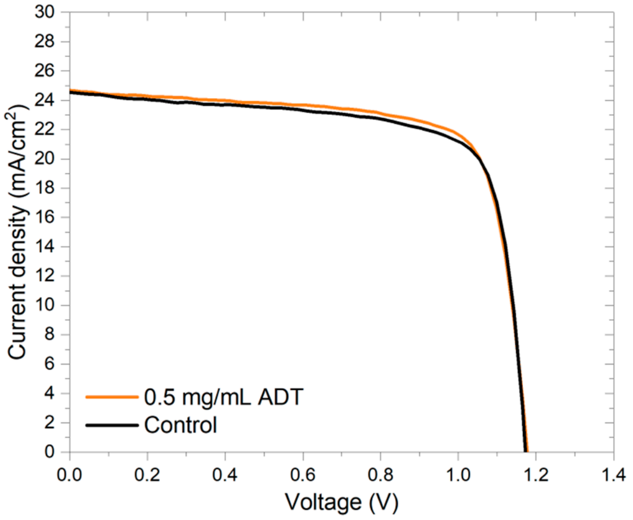

Figure S11. J–V curves of the champion control and 0.5mg/mL ADT-treated p-i-n perovskite solar cells measured under AM 1.5G illumination. The 0.5mg/mL ADT-treated device achieves a PCE of 21.71%, compared with 21.33% for the freshly prepared control.

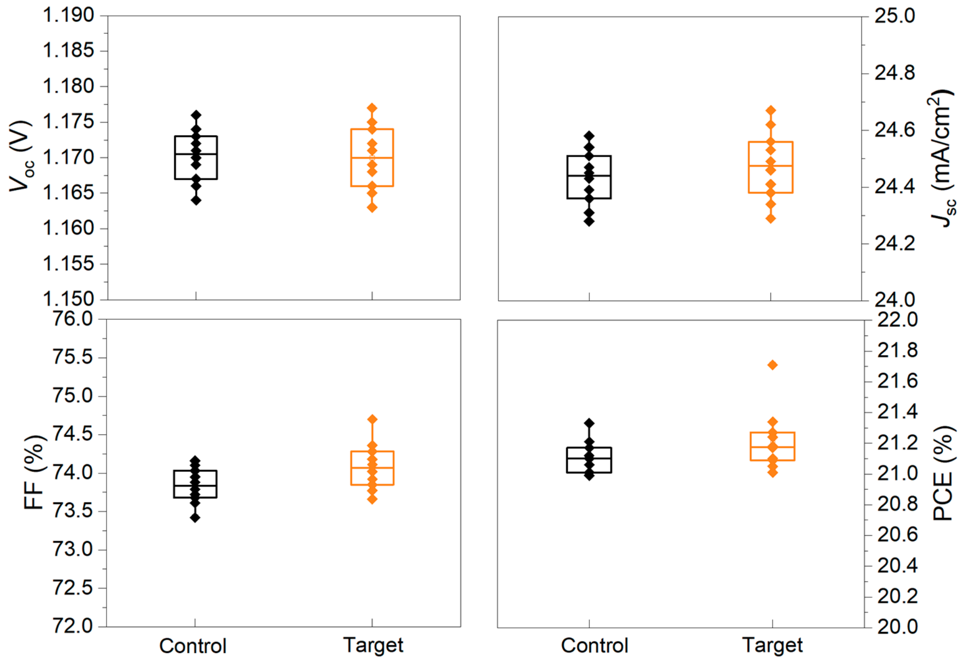

Figure S12. Photovoltaic parameters of control and 0.5mg/mL ADT-treated p-i-n perovskite solar cells. The 0.5mg/mL ADT-modified devices exhibit comparable open-circuit voltage (V_{OC}), short-circuit current density (J_{SC}), fill factor (FF) and PCE compared to the control devices.

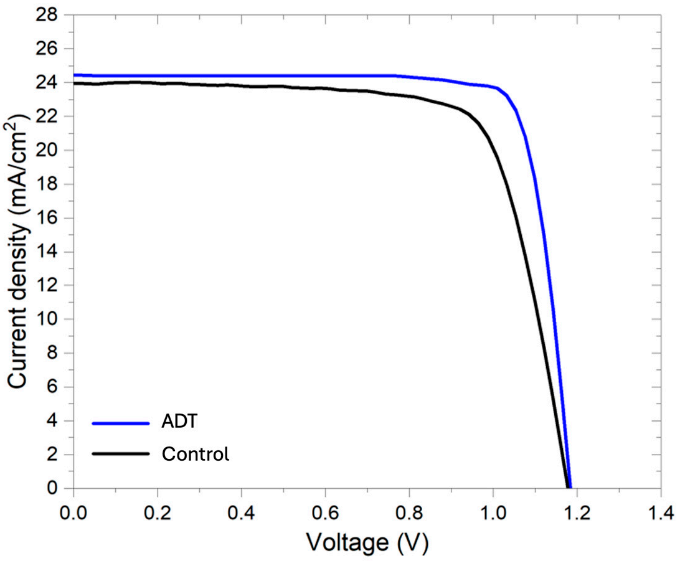

Figure S13. J–V curves of the champion control and ADT-treated n-i-p perovskite solar cells measured under AM 1.5G illumination. The ADT-modified device achieves a PCE of 23.91%, significantly outperforming the control device (20.86%)

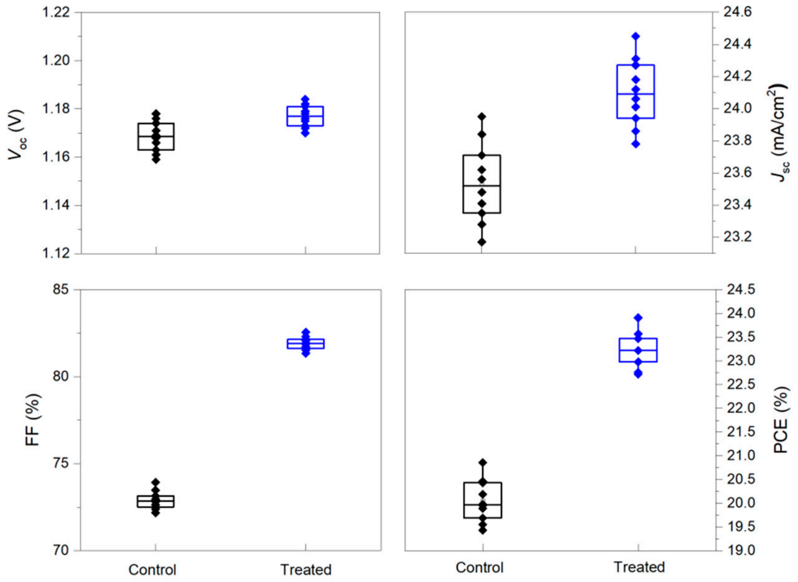

Figure S14. Photovoltaic parameters of control and ADT-treated n-i-p perovskite solar cells. The ADT-modified devices exhibit enhanced open-circuit voltage (Voc), short-circuit current density (Jsc), and fill factor (FF), leading to a significantly improved PCE compared to the control devices, demonstrating the effective passivation and interfacial optimization by ADT.

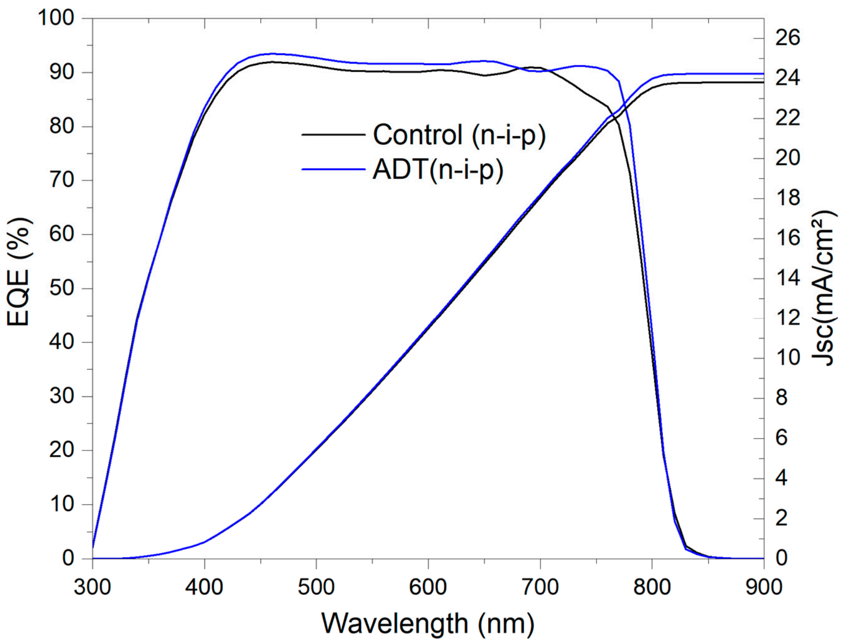

Figure S15. EQE spectra and integrated J_{sc} curves of the control and ADT-treated n-i-p devices.

Table S1. The mean ± standard deviation values for all photovoltaic parameters of both control and ADT-treated n-i-p devices.

Parameter	Control	ADT-treated
Jsc (mA cm^{-2})	24.098 ± 0.096	24.416 ± 0.088
Voc (V)	1.1575 ± 0.0032	1.1650 ± 0.0023
FF (%)	82.92 ± 0.43	83.12 ± 0.27
PCE (%)	23.13 ± 0.18	23.65 ± 0.13

3 References

1. Whelan, P.R.; Shen, Q.; Luo, D.; Wang, M.; Ruoff, R.S.; Jepsen, P.U.; Bøggild, P.; Zhou, B. Reference-Free THz-TDS Conductivity Analysis of Thin Conducting Films. *Opt. Express* **2020**, *28*, 28819, doi:10.1364/OE.402447.
2. Lin, R.; Gao, H.; Lou, J.; Xu, J.; Yin, M.; Wu, P.; Liu, C.; Guo, Y.; Wang, E.; Yang, S.; et al. All-Perovskite Tandem Solar Cells with Dipolar Passivation. *Nature* **2025**, *648*, 600–606, doi:10.1038/s41586-025-09773-7.
